# Sustainable colonization of Mars using shape optimized structures and in situ concrete

**DOI:** 10.1038/s41598-023-42971-9

**Published:** 2023-09-21

**Authors:** Omid Karimzade Soureshjani, Ali Massumi, Gholamreza Nouri

**Affiliations:** Department of Civil Engineering, Faculty of Engineering, Kharazmi University, Tehran, Iran

**Keywords:** Astronomy and planetary science, Engineering, Civil engineering

## Abstract

The major obstacle to Martian colonization is the mission cost which requires significant reduction. From the structural engineering point of view, importing materials and structural elements from Earth or massive excavations on the surface of Mars require an enormous amount of energy; thus, inflatable and under-surface structures as the main options for Martian colonization seem unrealistically expensive. Construction of affordable buildings onsite using only in situ sources may represent an ideal solution for Martian colonization. On the other hand, solar energy, at the early stage of colonization, would be the only available, practical, and low-cost energy source on Mars. Though, for sustainable and broad colonization, the energy required for construction and the construction cost should be minimized. Here, we propose three types of simple (relatively optimized), perforated, and algorithmic shape-optimized Martian structures to minimize the material and energy required for construction as well as the construction cost using only in situ resources. These structural forms can be considered remarkable steps towards sustainable structural construction and colonization on Mars. Also, these innovative structures were designed to minimize the tensile stress (maximize the compressive stress) and enable the use of in situ concrete. Our data indicate that compared to our previous study, the material and energy required for construction as well as the construction cost can be reduced by more than 50%. Acceptance criteria and limitations appropriate to the Martian environment, and desirable structural and material behaviors were defined to evaluate whether or not the behavior of a structure under the applied loads and conditions will be acceptable. To detect potential issues for onsite construction and evaluate the geometry of the models, a 1:200 3D model of the best structural form was printed.

## Introduction

Earth has experienced five massive extinction events where almost 85% of living species have become extinct in the past 500 million years^1^. Some scholars assert that we are now in the midst of the Holocene massive extinction where the increase in the human population and unchecked devastation of its resources will go beyond Earth’s carrying capacity to rejuvenate itself. Some scientists believe that human beings will be forced to seek out energy supplies from other sources such as planets and stars^2–4^. A multi-planetary life could be an appropriate answer to such concerns. Additionally, it could provide economic opportunities such as space tourism, new resources to exploit, exploration of new worlds and help humanity to reach its full potential^5,6^. Thus, multi-planetary life seems inevitable and Mars will most likely be our first destination.

With the increase in knowledge and data about Mars and its behavior, studies focusing on the colonization and construction on Mars have accelerated in the present century^7^. The Lunar/Mars Program Office at Johnson Space Center proposed a precast prestressed concrete structure with a diameter of 36 m and height of 22 m (dome-shaped) which tolerated the desired internal pressure for planetary use^8^. NASA studied suitable structural forms for human settlements on Mars for a total mission time of 919 days (224 days outbound from Earth, 458 days on Mars and 237 days of inbound to Earth)^9^. The structure was metal and cylindrical in shape with a diameter of 8 m that could tolerate the internal pressure difference. On such a mission, more than 20% of the mission cost would be for the structures/habitats to be imported from Earth.

Inflatable structures are among the most interesting types of structures proposed for life on Mars. These would be relatively lighter than steel structures and easy to pack and deploy. One of the best examples of inflatable structures is TransHab^10^.

The use of caves and glass domes as human habitats also have been studied. Caves are good for load bearing and could be sealed; however, their availability, size and conditions on Mars have not been ascertained. They may be a good option for the second stage of colonization^11^.

The export of structural elements, including inflatable structures, to Mars should be minimized because of its cost. It would be difficult to settle Mars without the use of in-place resources and onsite construction^12,13^. Mars is an elemental-reach planet. Onsite and remote observations by rovers, landers and orbiters show that there are several kinds of elements, salts, sulfates and compositions in the Martian soil (regolith) that can be used for crafting Martian concrete/cement^14,15^. Sulfur concrete, Mg- and Si-based concrete, Plaster of Paris (POP) and Geopolymeric cement are examples of Martian concrete that can be built using in-place sources^16^. But the common point between these concrete/cement is the brittle behavior and low tensile strength (compared to the compressive strength).

Because of the brittle behavior and low tensile strength of Martian concrete, as well as Martian structural loads, the design of structures on Mars using only in-place resources would be a challenge^13^. For this issue, Petrov and Ochsendorf proposed Martian structures that covered roughly 10 m of the regolith^17^. They tried to design a suitable permanent Martian base using in-place resources and a hybrid design. Kozicki assessed low-cost solutions for a Martian city that included the use of tunnels, caves, terraced villages, craters and tubular elements^18^.

Besides inflatable Martian structures, under-surface or near-surface structures have been of interest^16^. In such structures, the weight of the regolith shield opposing the structural internal pressure would keep the structure stable, making it possible to design structures using in-place Martian concrete. Such structures still would require massive excavation in some cases that should be considered in accordance with the harsh environment of Mars^11,19^.

A dome-shaped Martian structure using the multi-layer material MadFlex has been designed^20^ that uses Dyneema, a composite unidirectional laminate and carbon layers. This gives MadFlex high strength on one side and flexibility on the other side. Troemner et al. proposed and designed a dome-shaped 3D-printed Martian habitat^21^. This hybrid habitat, an inflatable structure with a 3D-printed shield, showed appropriate behavior under Martian environmental loads from wind, dust deposition, gravity and meteoroid impact.

Soureshjani et al.^13^ proposed a design load combination for which a Martian structure could be designed that would properly tolerate Martian structural loads. Probable Martian structural loads have been studied and calculated using valid data recorded by landers, rovers and orbiters. Three innovative Martian structures that required only in-place sources for construction have been designed and proposed by Soureshjani and Massumi^22^ that would show appropriate behavior under probable Martian structural loads. Based on the analysis, the proposed structures showed their superiority over traditionally proposed Martian structural forms, such as dome-shaped structures; however, the proposed structures were not optimized.

To colonize Mars, costs should be reduced by a factor of 50,000^6^. This means that in-place resources must be used to maximize cost-effectiveness. Thus, providing reliable, human-friendly, simple and relatively inexpensive Martian buildings is a challenge to such a mission. Previous studies have addressed this issue by proposing the use of inflatable, under-surface/near-surface and hybrid structures (inflatable structure with a regolith shield). When considering the number of structures required and their shipping cost (130 to 200 thousand dollars per kg), inflatable structures would only be feasible for one or two small Martian bases. Under-surface/near-surface and hybrid structures would require extensive excavation (up to 20 m, in some cases). Considering the decrease in performance and paucity of excavators on Mars compared to Earth (due to decreased gravitational acceleration), machine failure in the harsh environment and the complexity of construction, such structures would not be viable for a major construction effort^11,19^. Probably, the only remaining option to reduce costs is onsite construction using in-place concrete and resources^12,13,23^.

As previously pointed, the composition of the Martian regolith could enable the production of several types of in-place concrete and binders, but they have been shown to have brittle behavior and limited tensile strength^16,24^. Because of Martian structural loads and their interaction along with the brittle behavior and low tensile strength of in-place Martian concrete, no optimum and sufficiently robust Martian building/base has been proposed for construction using only in-place resources (Martian binders) to date. This is why previous studies have suggested inflatable, under/near surface and hybrid structures.

On the other hand, solar energy, at the first stage of colonization, would be the only available, practical and low-cost energy source on Mars. Minimizing the material required for construction (using shape optimization) parallel to using only in situ resources for construction (without any massive excavation) would lead to sustainable structural construction on Mars.

**Figure 1:**
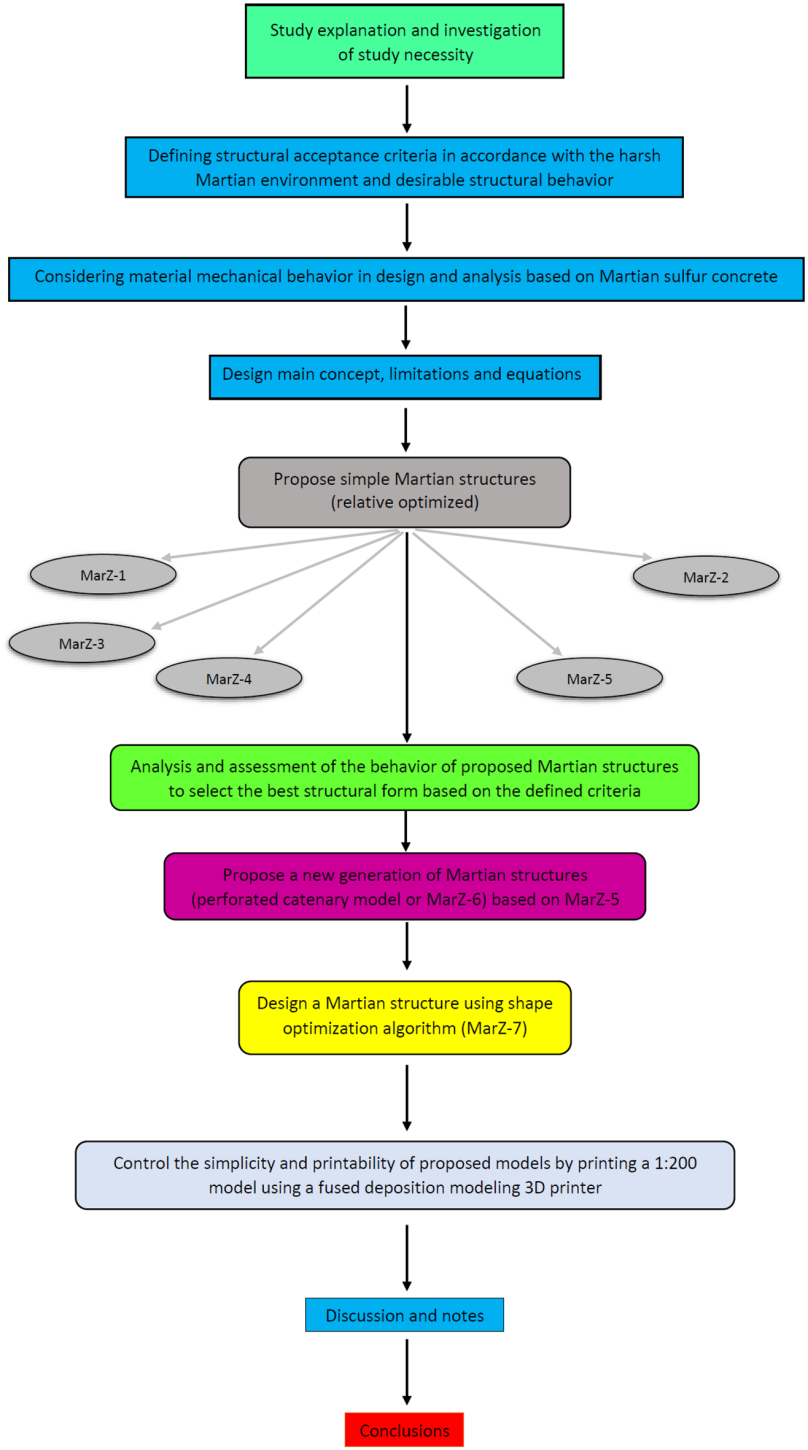
Flowchart of the research strategy.

We address this issue by introducing three groups of shape-optimized Martian structures. It has been tried to minimize the material required for construction as well as to energy required for construction using innovative forms, relative optimization and shape optimization algorithm. In addition to the use of only in situ Martian concrete for construction (These structures would be able to convert the tensile stress from Martian structural loading to compressive stress, minimize tensile stress, which would enable the use of in-place Martian concrete using rotated and reverse symmetric optimum parabolic fixed arches) and minimization of material required for construction, these structures could be a key for sustainable colonization in Mars. The proposed structures have been analyzed using implicit finite element (FE) analysis under probable Martian structural loads. Because of the harsh environment on Mars, acceptance and comparison criteria have been introduced to select the best structural system with the most appropriate structural behavior and less energy required for construction. Also, the printed 1:200 3D model of the best structure shows that the use of 3D printing of the proposed structures is possible because of the simple connections, circular shape and proper geometry. The proposed Martian structures can remarkably decrease the construction and settlement costs on Mars and make this dream come true. Figure 1 shows the research strategy of the current study.

## Results

### Proposed Martian structures

Five relatively optimized Martian structures with simple forms are presented below that have been generated, investigated and analyzed based on the structural concept of “Main concept, limitations and equation for design” section (using rotated and reverse symmetric optimum parabolic fixed arches to minimize tensile stress, which would enable the use of in-place Martian concrete), the acceptance criteria (“Defined structural acceptance criteria” section), material mechanical behavior (“Mechanical behavior of materials” section), limitations and equation (“Main concept, limitations and equation for design” section) to minimize the material and energy required for construction as much as possible (sustainable structures) using shape optimization. Two additional Martian structures (non-simple or structure with middle perforated layer and algorithmic shape-optimized) also have been proposed.

#### MarZ-1

**Figure 2:**
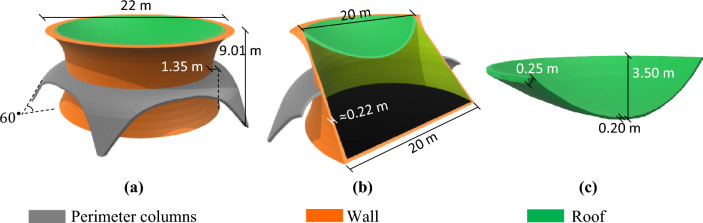
MarZ-1: **(a)** 3D view; **(b)** elevation view; **(c)** roof.

With a total height of 9.01 m and base and top radii of ${r}_{b}$ = 10.00 m and ${r}_{r}$ = 11.00 m, respectively, this model has a net internal volume of about 1741.22 m^3^. It consists of three main structural elements: the roof (${h}_{r}/{b}_{r}=0.17$, ${t}_{r}\approx 0.20\text{ m}$), perimeter wall (${h}_{w}/{b}_{w}=0.15$, ${t}_{w}\approx 0.22\text{ m}$) and five perimeter columns with thicknesses of 0.20 m at a 60° angle to the surface and a height of about 4.5 m. The columns control the perimeter stresses caused by the upward force of the internal pressure on the roof and the wall section reduction (due to the use of a rotated arch). These columns control the stress, provide appropriate stiffness at the middle of the wall and transmit loads to the surface (almost compressive behavior for columns). An extra perimeter edge has been designed at the top of the structure in order to control the concentrated stress at the roof-wall connection. Figure 2a–c show the details of MarZ-1.

It should be noted that according to “Main concept, limitations and equation for design” section and Eq. (3), ${h}_{r}/{b}_{r}$ is the ratio of the height to the length of the horizontal span of the roof arch, ${r}_{b}$ is the base radius, ${r}_{r}$ is the roof radius, ${t}_{r}$ is the roof thickness, ${h}_{w}/{b}_{w}$ is the ratio of the height to the length of the vertical span of the wall arch and ${t}_{w}$ is the wall thickness.

#### MarZ-2

**Figure 3:**
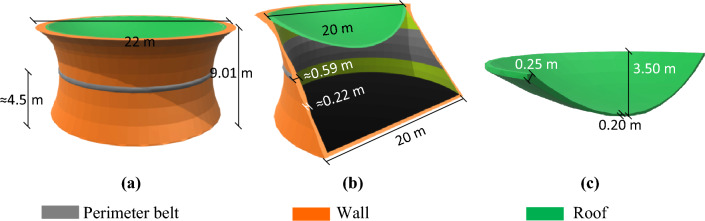
The MarZ-2: **(a)** 3D view; **(b)** elevation view; **(c)** roof.

MarZ-2 has a net internal net volume, total height and base radius that are similar to MarZ-1. The goal here has been to decrease the number of structural elements (decrease material required for construction) and present a simpler structural form than MarZ-1. MarZ-2 contains two main structural elements: the perimeter wall (${h}_{w}/{b}_{w}=0.15$, ${t}_{w}\approx 0.22\text{ m}$) and the roof (${h}_{r}/{b}_{r}=0.17$, ${t}_{r}\approx 0.20\text{ m}$, ${r}_{r}=10.00\text{ m}$). However, unlike MarZ-1, this structure uses internal and external belts at the middle of the wall (about 4.5 m) to control the in-plane stresses due to the upward force of the internal pressure on the roof and the wall section reduction. The concentrated stress at the roof-wall connection has been controlled as explained for MarZ-1 (“MarZ-1” section). Figure 3a–c shows the details of MarZ-2. It should be noted that, because of the internal belt, the internal net volume showed a 0.5% decrease (1732.22 m^3^) compared to MarZ-1.

#### MarZ-3

**Figure 4:**
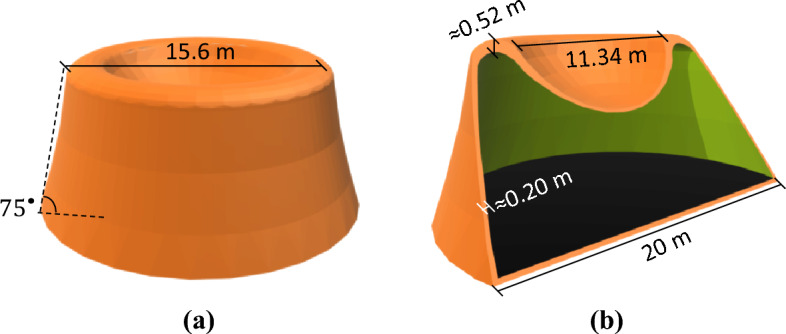
MarZ-3 model: **(a)** 3D view; **(b)** elevation view.

The MarZ-3 model has been designed as a one-piece integrated structure that simplifies construction (using a 3D printer) and avoids concentrated stress at the roof-wall connection. The total height, base radius and net internal volume of the structure are 8.63 m, 10.00 m and about 1700.11 m^3^, respectively. The perimeter wall is conical in shape at an angle of about 75° to the surface. Analysis shows that its conical geometry and roof perimeter ($${P}_{r})$$ that is smaller than the base perimeter ($${P}_{b}$$; about 0.60 $$\le {P}_{r}/{P}_{b}\le 0.90$$) improved the structural behavior and provided a uniform stress contour on the wall under Martian structural loads. Using relative optimization, $${P}_{r}/{P}_{b}\approx 0.66$$ was considered for this structure. A perimeter wall where $${h}_{w}/{b}_{w}\approx 0.07$$ and $${t}_{w}\approx 0.20\text{ m}$$ and a roof where $${h}_{r}/{b}_{r}=0.30$$ and *t*_*r*_ = non-uniform were considered. Figure 4a,b show the 3D and elevation views of MarZ-3, respectively.

The use of ${P}_{r}/{P}_{b}\approx 0.78$ for MarZ-3 improved the structural behavior and decreased the tensile stress (in-plane and out-of-plane) in the wall. This meant that no perimeter belt was required to control stress in the wall, thinner walls could be considered, and it was possible to consider a lower ratio of height to horizontal span length for the wall arch, which increased the net internal volume. The total height of the structure decreased compared to the MarZ-1 and MarZ-2, which will likely decrease the amount of material and energy required for construction (“Assessment of the behavior of proposed Martian structures” section). The optimum value for ${P}_{r}/{P}_{b}$ should be calculated case-by-case by considering the structural size, the ratio of the height to the length of the horizontal span of the wall arch, roof size, performance and efficiency of the structure as well as the architectural limitations, stress contours and the net internal volume.

#### MarZ-4

**Figure 5:**
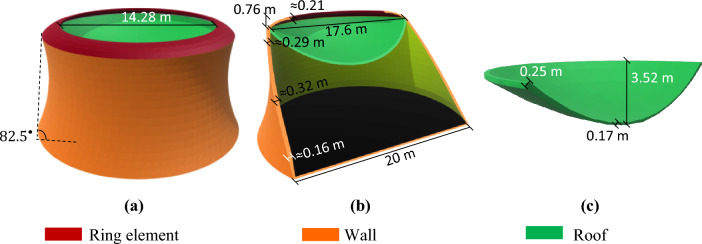
MarZ-4 model: **(a)** 3D view; **(b)** elevation view; **(c)** roof.

Similar to MarZ-2, the structure of MarZ-4 consists of two main elements: the roof and the wall^22^. The ratio of ${P}_{r}/{P}_{b}=0.88$ suggests that this model is a combination of MarZ-3 and MarZ-2 with an additional structural element (the ring) that has been employed to control the concentrated stresses at the roof-wall connection. The ring has been designed to behave compressive under Martian structural loads, increase the roof-wall connection stiffness, control the concentrated stress and decrease the undesirable strain. This makes it possible to control the stress and strain at the roof-wall connection using much less material than is required for MarZ-3 and MarZ-2. It would be a much more efficient way to control the roof-wall connection stress compared to previous models. If the ring element has been designed appropriately, it will intensively improve the structural behavior, increase its sufficiency and decrease the amount of material required for construction. The details of the ring element are shown in Fig. 5a,b.

The total height, base radius and internal net volume of MarZ-4 are 9.96 m, 10 m and 1713.70 m^3^, respectively. A perimeter wall where ${h}_{w}/{b}_{w}\approx 0.12$ and ${t}_{w}\approx 0.32\text{ m}$ and a roof where ${h}_{r}/{b}_{r}=0.20 \text{ m}$ and ${t}_{r}\approx 0.17 \text{ m}$ have been considered. The perimeter wall is conical in shape at an angle of about 82.5° to the surface. These sizes are in accordance with the relative optimization. The thickness of the wall changed from 0.16 to 0.32 min in accordance with the tensile stress required to control it. Figure 5a–c show the details of MarZ-4.

#### MarZ-5

**Figure 6:**
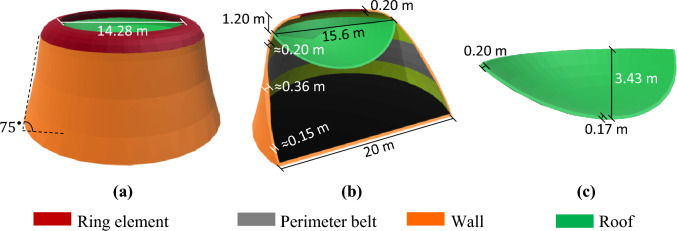
MarZ-5 model: **(a)** 3D view; **(b)** elevation view; **(c)** roof.

Assessment of the behavior of the proposed models showed that the ring element performed better at lower values of ${P}_{r}/{P}_{b}$ (compared to MarZ-4). Following relative optimization, the ring element performed best at ${P}_{r}/{P}_{b}\approx 0.78$. In this approach, the performance of the ring element was considered to be in accordance with the stiffness, material required, appropriate stress distribution and compressive behavior under Martian structural loads. In this model (MarZ-5), the ring element behaves completely compressive under Martian structural loads. Details of the ring element are shown in Fig. 6a,b.

MarZ-5 is conical in shape with a total height of 9.50 m and a net internal volume of 1698.08 m^3^. The wall was designed at an angle of 75° to the surface and has a geometry that is similar to that of MarZ-3. Because of the high stiffness and good performance of the ring element, the undesirable strain and stress at the roof-wall connection decreased remarkably and the concentrated stresses could be controlled with much less material. The appropriate shape of MarZ-5 makes it possible to choose a thinner roof and wall or lower the $h/b$ ratios compared to MarZ-1 through MarZ-4. The structural parameters of ${h}_{w}/{b}_{w}\approx 0.07$, ${t}_{w}\approx 0.2\text{ m}$, ${h}_{r}/{b}_{r}=0.22 \text{ m}$, ${t}_{r}\approx 0.17 \text{ m}$ were chosen in accordance with relative optimization. A perimeter belt with a maximum thickness of 0.36 m was employed at the middle height of the structure to control the perimeter in-plane tensile stresses caused by the internal pressure of the upward force on the roof and the section reduction. Figure 6a–c shows the details of MarZ-5.

#### Assessment of the behavior of proposed Martian structures

All the proposed models were circular in shape with a base area of 314 m^2^. Their net internal volumes were similar (less than 2% difference), which is in accordance with the RD dome of Kozicki and Kozicka^25^. From a structural behavior perspective, the models are comparable.

**Figure 7:**
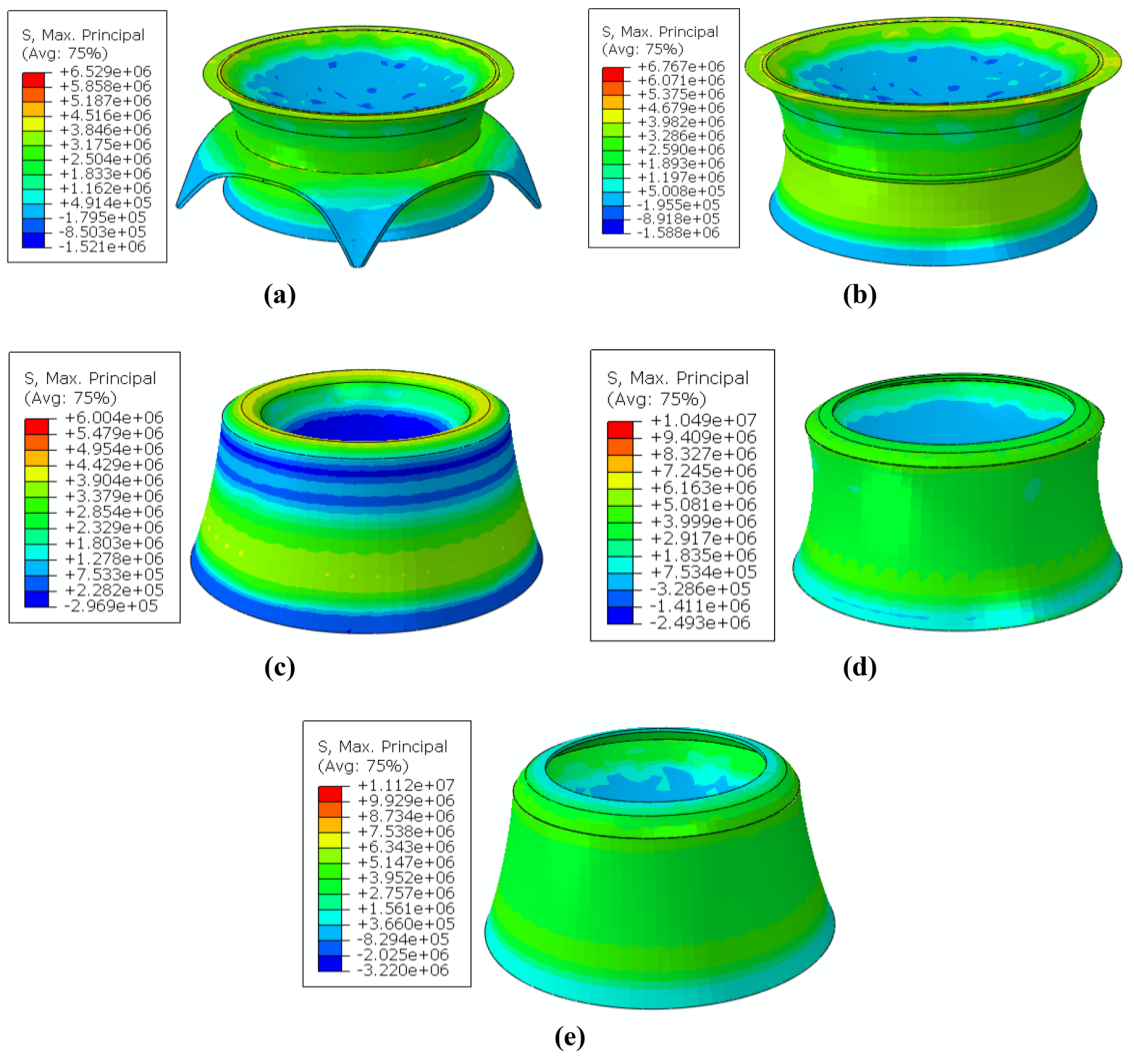
Behavior of proposed Martian structures under Martian structural loads (MPS): **(a)** MarZ-1; **(b)** MarZ-2; **(c)** MarZ-3; **(d)** MarZ-4; **(e)** MarZ-5.

Figure 7a–e show the maximum principal stress (MPS) contours of the proposed Martian structures under dominant Martian structural loads (internal pressure plus dead loads). In order to minimize the amount of material required for construction, an effort was made to utilize all the elastic tensile capacity of the sulfur concrete. This was achieved using relative optimization. All the MPS curves show completely elastic behavior under the tensile strength limit of the intended Martian sulfur concrete (about 4 MPa). This is reported in the supplementary information section (Supplementary Fig. 1). Thus, MarZ-1 to MarZ-5 showed either no plastic strain or no structural damage. Based on the defined structural acceptance criteria (“Defined structural acceptance criteria” section) and design limitations (“Main concept, limitations and equation for design” section), MarZ-1 to MarZ-5 are acceptable structural forms that offer stable and rational behavior under dominant Martian structural loads. Because of the relative optimization and suitable behavior of the arches, all the structures would show appropriate linear behavior on Mars. In Table 1 of the supplementary information section, more details about the structural behavior of each structure have been depicted.

Next, the amount of material required for the construction of the acceptable structures was investigated to determine the best structural model. The structure that requires the least amount of material for construction would be considered as the better model because it would require less energy to construct (“Defined structural acceptance criteria” section). Also, from the material required for the construction point of view, MarZ-5 required 221.01 m^3^ of concrete, which is less than for the other models (MarZ-1 through MarZ-4). For example, MarZ-5 requires almost 37% less concrete than MarZ-1 and 10% less than MarZ-4. The comparison between material required for construction of each structure is shown in the supplementary information section (Supplementary Fig. 2).

**Figure 8:**
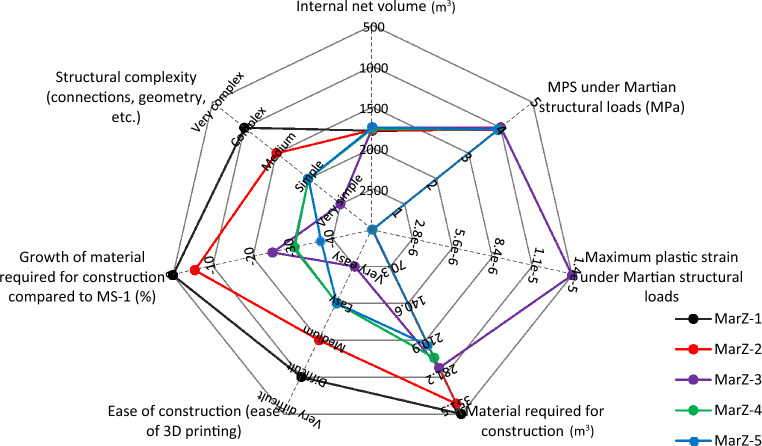
Comparison of multi-scale spider/radar chart in accordance with defined acceptance criteria and design limitations.

Indeed, parallel to stable and complete elastic behavior, MarZ-5 requires less energy for construction (less cost) and can be considered as the best Martian structure compared to MarZ-1 through MarZ-4. Its other advantages include uniform tensile stress contour, better architectural performance (because of a low ${h}_{w}/{b}_{w}$), completely compressive behavior of the ring element and ease of printing (because of its structural geometry). The multi-scale spider/radar chart (Fig. 8) compares the proposed Martian structures in accordance with the defined acceptance criteria (“Defined structural acceptance criteria” section) and design limitations (“Main concept, limitations and equation for design” section).

According to the construction process for a Martian structure (“Main concept, limitations and equation for design” section), the gravity load (dead load) should be applied first and then the internal pressure (as the dominant structural loads of a Martian structure). The structure must be stable during this loading process. Stability of the structures under the mentioned loading process is presented in Supplementary Fig. 3.

#### Structure with middle perforated layer

**Figure 9:**
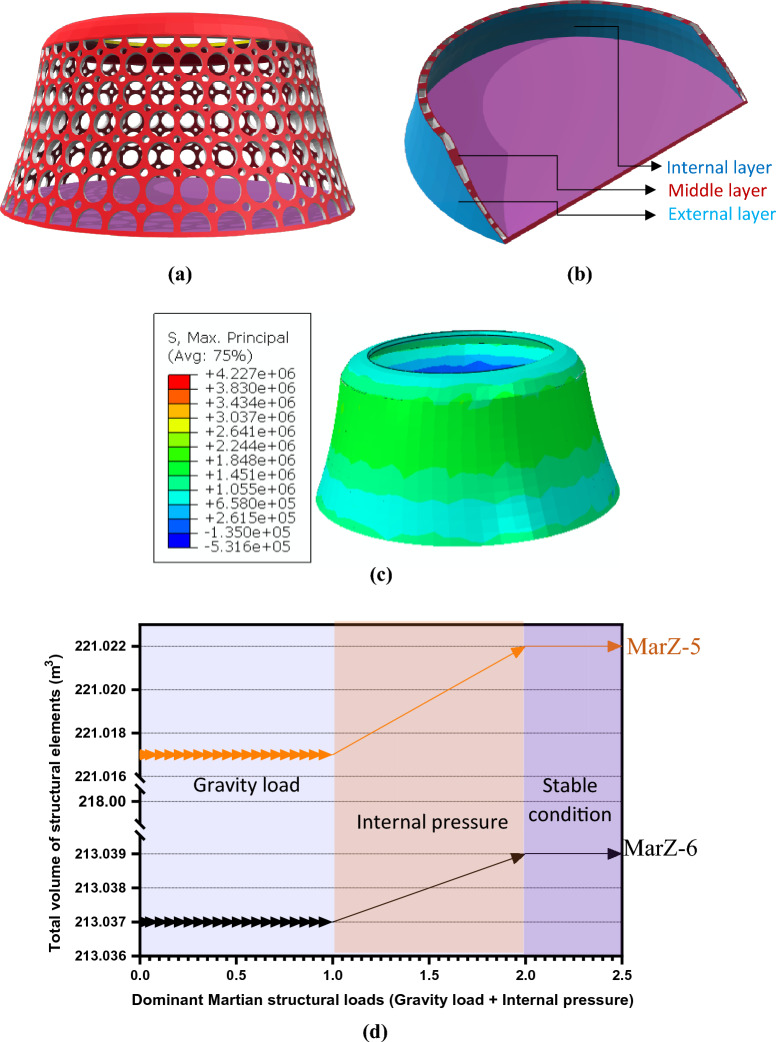
MarZ-6: **(a)** middle perforated layer; **(b)** external layer plus middle layer plus internal layer scheme; **(c)** MPS contour under Martian structural loads; **(d)** total volume of structural elements under Martian structural loads (volume stiffness).

The moment of inertia of an object depends on the perpendicular distance from the axis to the object’s center of mass^26^. It is possible to increase either the moment of inertia or stiffness of an element by increasing its dimensions (Eqs. 1, 2)^27^. With this in mind, a novel Martian structural concept (MarZ-6) with internal and external layers and a middle perforated layer was proposed and designed. The general form of MarZ-6 was similar to MarZ-5, which was selected as the best Martian structural form (“Assessment of the behavior of proposed Martian structures” section; Figs. 7, 8). Figure 9a–c show the details of the MarZ-6 model. The perforated layer (middle layer) contains circles at the top with diameters of 0.5 and 0.16 m placed such that the minimum distance between the circles remained constant (0.20 m). Because of the conical shape of the structure, the diameter of the circles was increased at an appropriate ratio as related to ${P}_{r}/{P}_{b}$ and the height. A minimum distance of 0.20 m was maintained between circles.

1 $${I}_{x}={\int }_{A}{y}^{2}dA,$$

2 $${I}_{y}={\int }_{A}{x}^{2}dA,$$

where ${I}_{x}$ and ${I}_{y}$ are the moments of inertia about the *x* and *y* axes, respectively, ${y}^{2}$ and ${x}^{2}$ are the distances from the $x$ and $y$ axes at any point, respectively, and $dA$ is the equation describing the width of the shape at any given value of $y$ or $x$ at which $y$ or $x$ changes.

Octagonal, hexagonal and diamond-shaped patterns were investigated and analyzed. Because of the concentrated tensile stress at the corners, these patterns did not qualify and showed inappropriate behavior under Martian structural loads. Thus, the circular shape, with no corners, provided the best behavior for the internal layer under Martian structural loads. A pattern based on circles thus was selected for the middle layer of MarZ-7. The Supplementary Video 1 shows a 3D view of the middle perforated layer.

To increase the stiffness of the wall, three layers with a total thickness of ${t}_{w}\approx 0.30\text{ m}$ (0.05 m external layer plus 0.20 m middle perforated layer plus 0.05 m internal layer) was considered for MarZ-6. The thickness of the MarZ-6 wall was about 50% more than that for MarZ-5. However, the middle perforated layer was designed to decrease the total concrete required for construction.

Analysis indicated that MarZ-6 showed acceptable behavior under Martian structural loads based on the structural acceptance criteria (“Defined structural acceptance criteria” section) and design limitations (“Main concept, limitations and equation for design” section). In this model, the MPS distribution (MPS contour) was similar to MarZ-5 (Fig. 7e, 9c). Thus, the amount of concrete required for construction and volume stiffness were considered as determinative parameters for the comparison of the MarZ-5 and MarZ-6 models.

The MarZ-6 requires 213.03 m^3^ of concrete for construction, which is 3.61% less than for MarZ-5. This means a decrease in construction energy, which will decrease construction costs. Figure 9d shows the changes in the volumes of the structural elements for MarZ-6 and MarZ-5 and the volume stiffness under dominant Martian structural loads. It can be seen that the volume stiffness of MarZ-6 is about twice that of MarZ-5. Similar results can be seen for the MPS contours of the models (Figs. 7e, 9c). This indicates that not only does MarZ-6 require less concrete for construction (− 3.61%), but it also provides twice the volume stiffness compared to MarZ-5. In other words, the perforated concept used for MarZ-6 can cut in half the amount of concrete required for construction compared to MarZ-5. This could lead to a decrease in the energy required for construction and in the mission costs.

#### Use of a shape optimization algorithm for the design of a Martian structure

Shape optimization is used at the end of a design procedure when the general shape and components of a model have been determined. Previous sections have described the general shape and geometry of the proposed Martian structures on which shape optimization can be used. The use of an appropriate shape optimization algorithm could produce a new Martian structure (MarZ-7) that shows improved behavior compared to the previously proposed models.

Shape optimization was done using an iterative algorithm that refines, modifies and repositions the model by moving the surface nodes to reduce concentrated stresses according to the defined limitations and goals^28,29^. The general purpose is to achieve stress homogenization. Using Python script and Abaqus, the following limitation, goal and law were used in accordance with the defined structural acceptance criteria (“Defined structural acceptance criteria” section) and limitations (“Main concept, limitations and equation for design” section) for shape optimization:

- *Optimization goal* Minimization of MPS is the goal of every optimization iteration until reaching linear behavior under dominant Martian structural loads.
- *Optimization limitation* The material volume was limited to 215 to 270 m^3^ (about 25% change).
- *Law* Optimization will end if the change in MPS between the current step and the previous step is below 0.02%.

**Figure 10:**
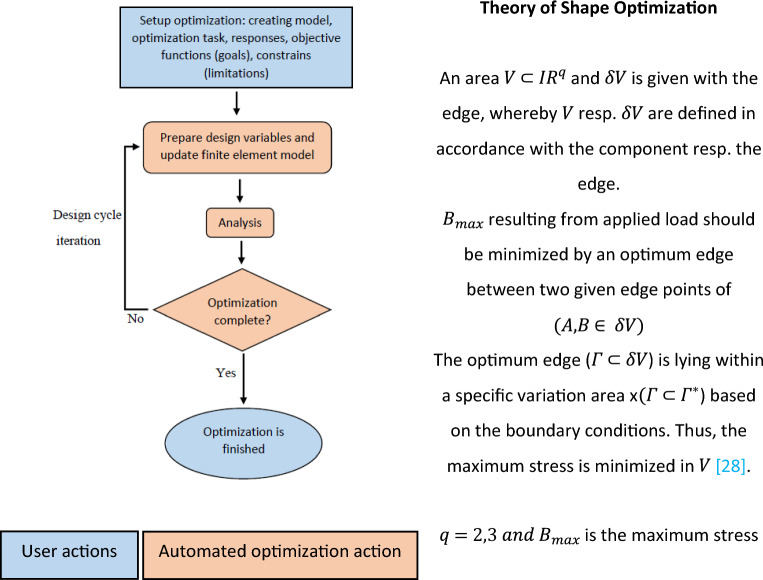
Shape optimization flowchart and theory.

Figure 10 shows the optimization flowchart and basic expressions^28–31^.

**Figure 11:**
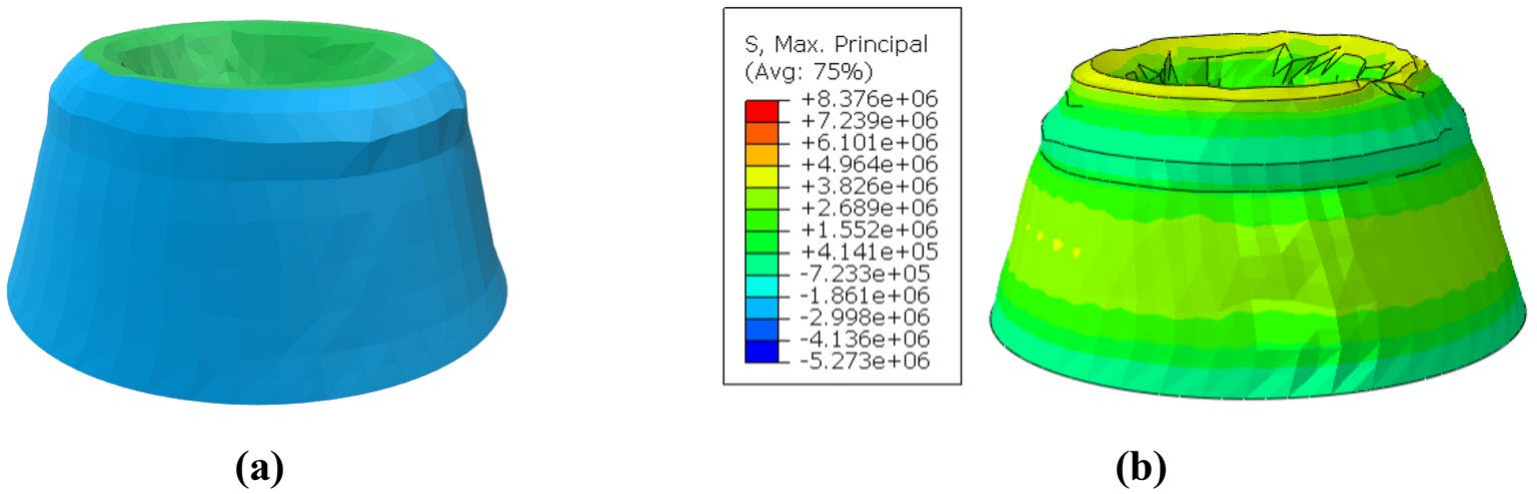
Shape optimization: **(a)** MarZ-7; **(b)** MPS contours under Martian structural loads.

The MarZ-5 model that was selected as the best-proposed model (“Assessment of the behavior of proposed Martian structures” section, Fig. 8) was used for shape optimization. Figure 11a,b show the optimized Martian structure (MarZ-7). It should be noted that the ring element was removed for shape optimization to allow the algorithm to propose a solution that could control the stress in the wall-roof connection. Also, both MPS minimization and strain minimization (to reach elastic strain) were considered as separate optimization goals. As MPS minimization showed better results, it was selected as the optimization goal. Refer to the supplementary information section (Supplementary Fig. 4) for optimization process and convergence.

Figure 11b shows that the MPS was lower than the tensile strength limitation (3.92 MPa); thus, the behavior of MarZ-7 was linear (no plastic strain and damage). Based on the structural acceptance criteria (“Defined structural acceptance criteria” section) and design limitations (“Main concept, limitations and equation for design” section), MarZ-7 is an acceptable structure.

Figure 11b shows that the shape optimization algorithm considered an extra thickness at the wall-roof connection to control concentrated stress. As stated before, the MarZ-5 model uses a ring that exhibits completely compressive behavior under structural loads and is more efficient.

MarZ-7 requires 14.59% more concrete for construction than MarZ-5, which translates into more energy and greater costs. Additionally, the geometry of MarZ-7, chiefly in the wall-roof connection, makes its geometry difficult for construction using a 3D printer. Thus, the MarZ-5 and MarZ-6 models were considered to be the best models.

#### Conceptual construction (3D printing)

Martian structures should be very simple to be built onsite and no humans should be required for the actual construction work. These structures should be built on Mars by means of automated onsite construction. All of the proposed Martian structures use simply fixed connections and are circular in shape to make them suitable for construction using 3D printing techniques, such as the CC technique^32^.

To reveal possible problems that may arise during construction, a 1:200 model was printed using a fused deposition modeling 3D printer. This type of 3D printer works almost similar to the CC technique. The MarZ-5 model was printed in 2 h and 9 min as an integrated model with the low detail-fast print option. Supplementary video 2 shows the printing process.

The print procedure showed that supports were needed for printing the roof. Internal supports that were equal to 3% of the internal volume were deemed appropriate. As it would not be possible to use such supports for an actual Martian structure, an inflatable formwork is suggested for construction. These types of formworks have been used previously on Earth. Inflatable formworks are used by the construction industry chiefly for pipe construction and casting of prefabricated structures^33^. An inflatable formwork could be used for numerous Martian structures. Because of the Martian atmosphere, inflating this tool would be simple. Also, sulfur concrete would be fast-curing concrete on Mars, which would make the use of an inflatable formwork practical.

**Figure 12:**
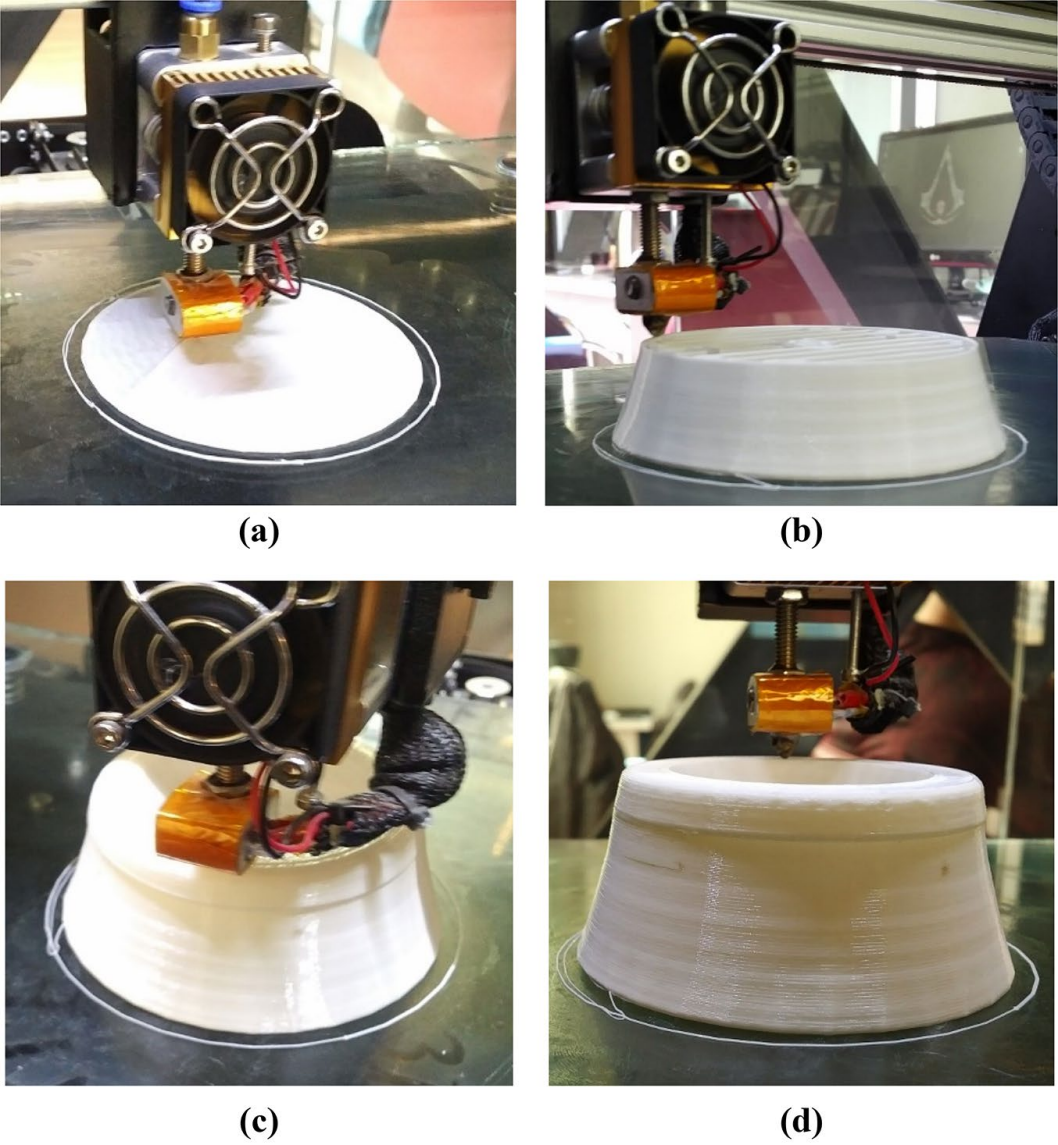
1:200 3D printing model MarZ-5: **(a)** foundation; **(b)** wall; **(c)** roof; **(d)** ring element.

In a nutshell, by considering the presented 3D printing process, simple geometry, simple fixed connections and circular shapes for the proposed structures, it is expected that the construction process would proceed without problems on Mars. Figure 12a–d shows the 1:200 3D printed MarZ-5 model.

It should be noted that this study proposes Martian sulfur concrete (“Mechanical behavior of materials” section) for onsite construction on Mars using an appropriate 3D printing construction method such as the CC technique. The very main concept of the CC technique and fused deposition modeling 3D printers (used for this section) are almost similar. Also, based on the studies, it is possible to construct high-detail full-scale structures using sulfur concrete and the CC technique^32,34^. However, for a comprehensive construction validation more studies using a full-scale structure, Martian concrete (based on the simulated Martian soil), simulated Martian conditions, and an appropriate 3D printing construction method (such as the CC technique) would be required.

## Discussion

Multi-planetary life is a possible solution to concerns that will affect future human generations. These include global warming, Holocene extinction, energy demand, protect the future of the human race and increases in the human population that will exceed Earth's capacity. Multi-planetary life can provide new economic opportunities such as space tourism and access to new resources. In this regard, Mars is a possible first destination for humans.

A major obstacle to the settlement of Mars is the high mission cost. Current solutions for Martian buildings and settlements, such as inflatable, under-surface and hybrid structures, are not cost-effective as they generally are based on the massive import of structural elements from Earth at a prohibitive cost. Additionally, it is not possible to colonize Mars using only inflatable structures. Onsite construction using only in-place resources and Martian concrete is a possible solution. However, the Martian structural loads that cause high tensile stress in a structure and the low tensile strength of Martian concrete remain a challenge.

On the other hand, solar energy, at the first stage of colonization, would be the only available, practical and low-cost energy source on Mars. By considering the thin atmosphere of Mars (positive effect) and distance from the Sun (negative effect), this energy would be almost 40% less than what we have on Earth. Minimizing the material and energy required for construction (using shape optimization) parallel to using only in situ resources for construction (without any massive excavation) may lead to sustainable structural construction and colonization on Mars.

Three types of innovative Martian structural forms of simple (relatively optimized), perforated and shaped optimized (based on the appropriate shape optimization algorithm) featuring symmetric optimum parabolic rotated and reverse fixed arches were proposed. These structures minimize tensile stress to decrease the energy required for construction as well as enable the use of in-place concrete. Such structures can remarkably reduce the energy and material required for construction parallel to eliminate the need for imports of structural components from Earth and could substantially reduce the cost of settlement construction and lead to sustainable colonization on Mars. The main results of our investigations are:

Structural damage (small cracks) on Mars can cause air leakage, freezing because of the humid internal environment of the structure and the atmospheric temperatures, expansion and, finally, collapse of the structure. Thus, considering the hazardous Martian environment and brittle behavior of Martian concrete and binders, it is recommended that Martian structures that are constructed using only Martian concrete should be designed based on linear behavior (no damage).

Considering the mechanical behavior of the Martian concrete, the Martian environment and desirable behavior for structures built on Mars, structural criteria should be defined to govern the behavior of Martian structures under Martian structural loads.

The acceptance criteria should include the maximum tensile stress (MPS), material required for construction, volume stiffness, plastic strain and net internal volume of a structure. These have been proposed in accordance with the brittle behavior of the in-place Martian concrete, energy required for construction, mission cost, absence of structural damage (linear behavior), a human-friendly design and the hazardous environment of Mars.

Generally, Martian concrete exhibits limited tensile strength and relatively appropriate compressive strength. The approach proposed for Martian structures in this study is the use of rotated and reverse symmetric optimum parabolic fixed arches to minimize the tensile stress under Martian structural loads and enable the use of in-place concrete. The proposed structural forms can decrease or eliminate the import of structural elements from Earth, enabling onsite construction on Mars and substantially decrease colonization costs.

The approaches used for the proposed structural forms are the minimization of the tensile stress and the maximization of compressive stress. Thus, these structural forms will be suitable for all types of Martian binders and concrete, such as extraterrestrial regolith biocomposites (AstroCrete), ice, Mg-based concrete, plaster, OPC, etc.

Symmetric optimum parabolic fixed arches showed the best performance and efficiency at a specific range of *h/b*. Considering the proposed structural forms, this ratio should be selected in accordance with the stiffness provided by the arches, net internal t volume of the structures, their efficiency and total size, the concrete required for construction, the possibility of 3D printing, structural behavior, interaction of loads and a human-compatible design.

The interaction of structural loads, such as the upward force on the roof by internal pressure and its effect on the walls, means it would not be possible to design a structural form that exhibits only compressive behavior under Martian structural loads. Thus, the structural forms have been designed to minimize tensile stress through relative optimization.

Limitations should be considered for the design of Martian structures. These limitations could be a circular shape for ease of printing and architectural and internal efficiency. The total height of the structures should make 3D printing and construction practical, provide an appropriate net internal volume, use simple (fixed) connections and avoid high *h/b* ratios. They should include construction and geometry integrity and simple geometry and appropriate thicknesses for the structural elements (wall and roof).

The proposed structures showed appropriate behavior under the worst-case scenarios and loading that would occur under actual conditions (stability under gravity (dead) loading followed by gravity (dead) load plus internal pressure.

Analysis showed that one important parameter that can improve the structural behavior under Martian structural loads, as well as a decrease in the amount of material required for construction is the ratio of the roof perimeter ($${P}_{r})$$ to the base perimeter ($${P}_{b}$$). In this case, the structures showed the best performance at 0.60 $$\le {P}_{r}/{P}_{b}\le 0.90$$. This parameter should be calculated case-by-case.

To control the concentrated stress in the wall-roof connections and increase stiffness (strain control) under Martian structural loads, a control element (ring element) in the form of an incomplete dome has been used. This element exhibited compressive behavior and showed good performance under structural loads which improved the behavior of the structure. With the use of this element and the increase in stiffness in the connection, it was possible to use a roof with a lower thickness or lower *h/b* ratio. This would decrease the amount of material required for construction.

Assessment of the behavior of the proposed structures under probable Martian structural loads showed that all of them were sufficient in accordance with the defined acceptance criteria and limitations. MarZ-5 showed the best behavior as well as the lowest amount of material required for construction. Aside from the better structural behavior, this structure required about 50% less material and energy for construction as well as the construction cost compared to MarZ-1.

To increase the stiffness of the wall and improve the behavior of the structure under Martian structural loads, a structure with middle perforated layer (MarZ-6) was designed that was based on MarZ-5. The MarZ-6 wall consists of three layers which are 10 cm thicker than that for MarZ-5 and uses a perforated pattern for the middle layer with a thickness of 0.20 m. This approach decreased the amount of material required for construction and improved the structural behavior. Analysis showed that MarZ-6 required 3.61% less material for construction and had about twice the volume stiffness compared to MarZ-5.

Shape optimization based on MarZ-5 (without a ring element) using an appropriate algorithm (Python script) was performed in increments to generate a new optimized structural model (MarZ-7). For optimization, minimization of the MPS and volume of materials (within a specific range) were considered as the goal and the limitations in accordance with the defined acceptance criteria (sec. 2), respectively. Considering the amount of material required for construction and the structural behavior under Martian structural loads, MarZ-7 was not necessarily a better structural form than those of MarZ-5 and MarZ-6.

## Methods

### Defined structural acceptance criteria

Appropriate criteria should be defined to evaluate the behavior of a structure under the specific applied loads and conditions^35^. Martian structures require comprehensive acceptance criteria that should be defined in accordance with the material properties, atmospheric conditions, environment of Mars and the desired behavior of the structures. In the harsh environment of Mars, structural damage at any level in structures made of in-place concrete could lead to disaster for humans. For example, a crack in a structure could lead to air leakage and freezing caused by the low temperature and moisture inside of the structure, followed by crack expansion and, ultimately, structural instability. Generally, Martian concrete exhibits brittle behavior (limited ductility) and has a relatively low tensile strength^13,16,24^. The approach of this study has been to design structures that show linear behavior (no damage) under Martian structural loads. The energy required for construction on Mars is also important; thus, the acceptance criteria should be based on related conditions and concerns.

**Figure 13:**
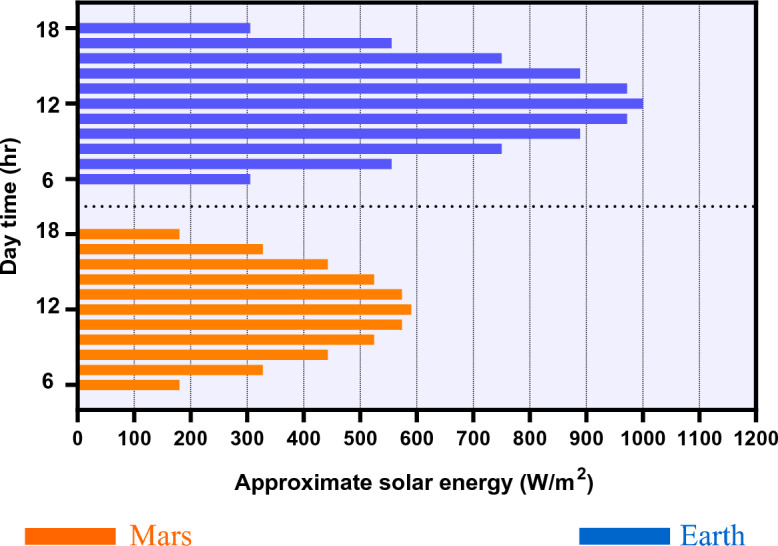
Approximate solar energy based on the parabolic equation.

Additionally, these criteria can be used for the comparison and optimization of structures to determine and design the best structural system for a structure on Mars. In this regard, the maximum principal stress (MPS), the energy required for construction, volume stiffness and maximum plastic strain should be determined as the acceptance and comparison criteria for introducing a reasonable Martian structure to be built onsite using only in-place Martian concrete. In the current study, the structural acceptance and comparison criteria were determined to be as follows:

- Martian concrete exhibits brittle behavior and relatively low strength under tension^13,16,24^. The MPS represents the greatest tensile stress and is an appropriate parameter for securing a safe design in structures built from brittle materials, such as those examined in this study^36,37^. It is possible to prevent structural damage (especially cracks) by controlling the MPS to provide a safe design. Additionally, MPS is a good parameter for relative and shape optimizations.
- This study proposes that Martian buildings should be constructed only of Martian concrete. As the geometry and concept of such structures will be almost similar, the concrete required for construction can be considered as the energy required for construction. In other words, as the amount of material required for construction increases, the energy required for construction also will increase. In the first stages of settlement construction, solar energy will be the most available source of energy^38^. It is doubtful that sources such as geothermal and nuclear power will be available in the early stages of settlement^39^. The maximum solar irradiance on Mars is roughly 43% of that on Earth (430 to 590 W/m^2^ versus 950 to 1371 W/m^2^, respectively)^40^. This means that the amount of concrete required for construction or energy required for construction should be considered as a comparison criterion for the mission cost. Like the MPS, the concrete required for construction is a good parameter for relative and shape optimizations. Figure 13 uses a parabolic equation to compare the approximate solar irradiance on Mars and Earth during a sunny summer day.
- The volume stiffness is a suitable parameter for comparison of the performance and behavior of different pressurized Martian structures having similar internal volumes and structural loads. The volume stiffness determines how strongly a solid object or structure can resist a change in volume^41^. In this study, this parameter has been defined as an increase in the total volume of structural elements under internal pressure (1 atm).
- The plastic strain is a valuable parameter for determining structural damage in brittle materials like concrete^42^. Unlike Earth structures, on Mars, structural damage at any level could lead to disaster^11,13,18,22^. Because of the brittle behavior of Martian concrete, the harsh environment of Mars and the susceptibility to damage to Martian structures under probable Martian structural loads, the plastic strain has been considered as a structural acceptance criterion. From this perspective, no plastic strain (damage) can be allowed for Martian structures.
- The net internal volume also is a structural acceptance criterion. For purposes of reasonable comparison, the net internal volume of all proposed models was considered to be similar. The volume of the Kozicki and Kozicka residential dome (RD dome) with a net internal volume of about 1705 m^3^ was considered as the reference volume^25^. There was less than 2% difference in the net internal volumes of all proposed Martian structures. The base area of the proposed models was considered to be 314 m^2^, which is the size of the RD dome (diameter = 10 m).

### Mechanical behavior of materials

As for structural loading, the mechanical behavior of the materials that make up a structure are important parameters for analysis. The aspects involved in the selection of building materials for a Martian structure to be built onsite using in-place concrete included the availability of raw materials, strength, durability, stress–strain behavior, curing time, permeability and ability to use 3D printers, etc.

**Figure 14:**
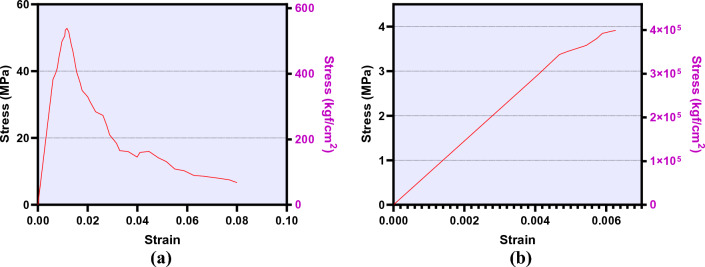
Average mechanical behavior curves of simulated Martian sulfur concrete under: **(a)** compression; **(b)** tension.

Mars is a sulfur-rich planet. Observation indicates that, on average, 2.48% (wt) of Martian soil is composed of sulfur^43,44^. The production of sulfur concrete on Mars is not complex and would require less energy than other binder types, such as ordinary Portland cement (OPC) or plaster, on Mars. Sulfur concrete is in a rhombic phase in the Martian environment and is waterless. It can be used in 3D printers, is workable in the Martian atmosphere and shows appropriate durability under the highly variable temperature of Mars^16,24,32,45^. This makes sulfur concrete a good choice for construction on Mars. The current study was based on the properties and mechanical behavior of sulfur concrete. The average mechanical behavior of simulated Martian sulfur concrete (composed of 50% (wt) Martian regolith and 50% sulfur) as introduced by Wendner et al.^22,24^ was considered for the current study. Figure 14a,b provide the average stress–strain curves of the intended Martian sulfur concrete. Additionally, Young's modulus (6.5 GPa) and Poisson's ratio (0.18) that are required for linear analysis were calculated in accordance with the stress–strain curve and experimental tests^22,24,46^.

It should be noted that by considering the similarity of the behavior of Martian concretes (brittle behavior and limited tensile stress) and the linear behavior of the proposed Martian building, the proposed structural forms are also suitable for other Martian concrete/cement and binders such as extraterrestrial regolith biocomposites (AstroCrete), POP, ice, Mg-based concrete, plaster, OPC, etc.

### Main concept, limitations and equation for design

The present study proposes seven Martian structures that have been designed and generated using rotated and reverse symmetric optimum parabolic fixed arches. Arches have the ability to convert any vertical load into a compressive load along the arch direction^47,48^. Considering the dominant Martian structural loads (internal pressure and dead loads), it is possible to minimize the tensile stress using these arches and innovative design forms^22^. With the low tensile strength and brittle behavior of Martian concrete, these structural forms are key components for the design of low-cost Martian structures using only in-place sources onsite.

The symmetric optimum parabolic fixed arches were calculated using Eq. (3)^47,49^. This equation provides an optimum arche shape that behaves completely compressive under applied vertical loads.

3 $$y=h\left(1-\frac{4{x}^{2}}{{b}^{2}}\right),$$

where *h* is the height of the symmetric arch, *x* and *y* are the cartesian positions of the arch and *b* is the horizontal span length.

**Figure 15:**
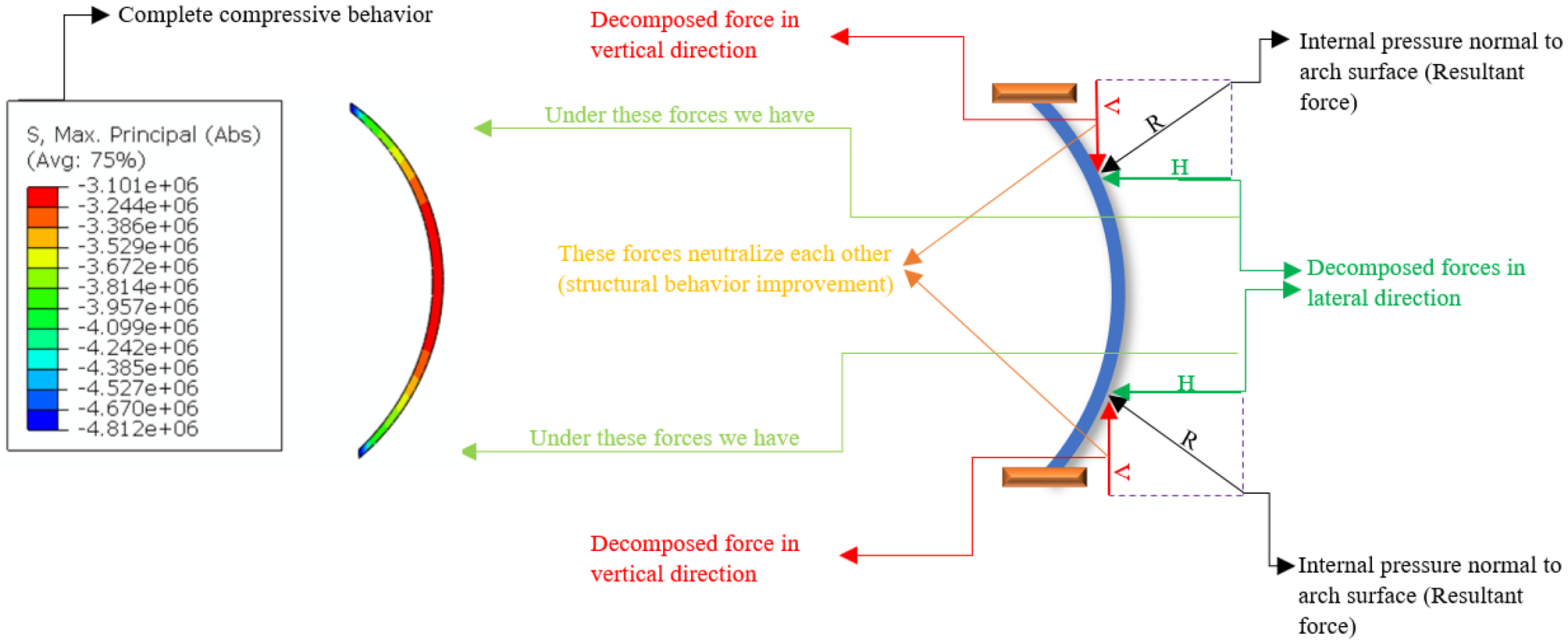
Rotated symmetric optimum parabolic fixed arches and decomposed forces under a normal load.

**Figure 16:**
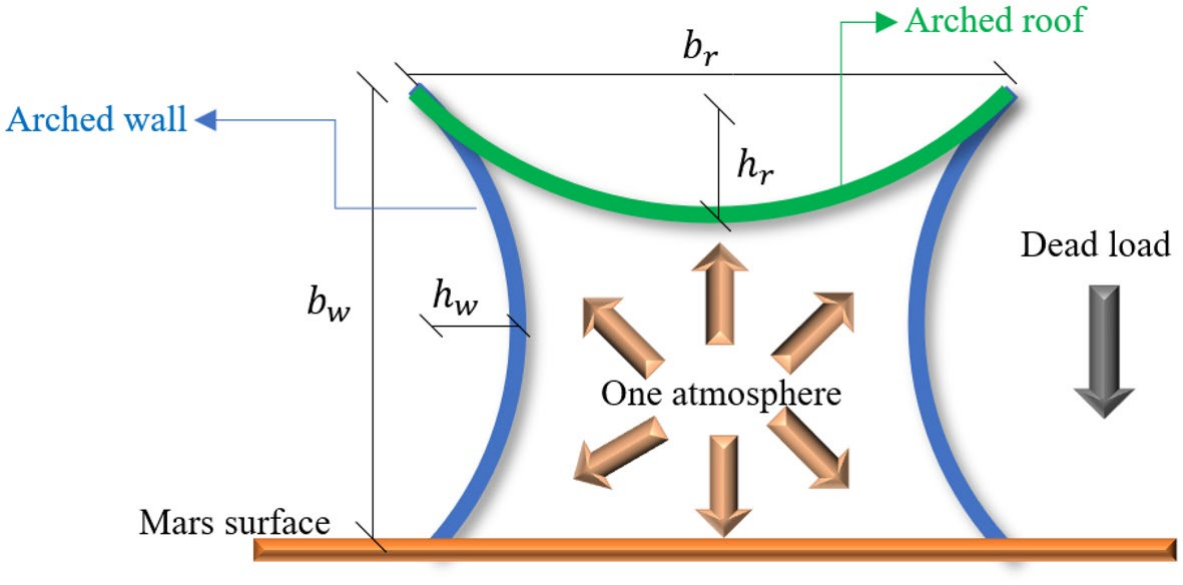
Main 2D concept of proposed Martian structures under dominant Martian structural loads.

Figure 15 shows a rotated 2D symmetric optimum parabolic fixed arch under a load, such as internal pressure that is normal to the surface, and the decomposed forces for an arch calculated in accordance with Eq. (3). The MPS contour of the arch is shown in the figure. It is the basic mechanism for the initial concept shown in Fig. 16 to minimize tensile stress. As seen, the arch exhibits compressive behavior.

Because of the interaction and diversity of Martian structural loads, the structural geometry that employs arches that exhibit complete compressive behavior is difficult. In other words, designing a Martian structure with compressive behavior is impossible.

The dominant Martian structural loads are dead loads at an internal pressure of 1 atm^13^. Because of the low tensile strength and relatively high compressive strength of Martian concrete, the arches should provide a geometry to minimize tensile stress under the dominant structural loads. Figure 16 shows a 2D elevated view of the proposed Martian structures under the dominant Martian structural loads considered in the present study (design main concept). The arches have been positioned such that they decrease the tensile stress and increase the compressive stress to enable the use of in-place concrete.

In consideration of the mechanical behavior of in-place concrete, the interaction of Martian structural loads and structural geometry, performance and design efficiency, some design limitations should be considered as follows:

- Studies have shown that the ratio of arch height to horizontal span length (*h/b*) has a major effect on the critical vertical load capacity of the intended arches^47,48^. Considering the structural concept used in this study (“Main concept, limitations and equation for design” section, Eq. 3), the optimum range of *b/h* for these kinds of arches for walls and roofs should be chosen according to the structural efficiency, performance, structural size, behavior and stability of the structure as well as the possibility and material required for construction. A high value for this ratio could increase the stiffness but could make the construction, overall stability and efficiency of the structure difficult to realize^49^.
- As the goal is the design of stable Martian structures using only in-place concrete, it should be possible to print the proposed structures using common 3D printing methods such as Contour Crafting. This means that the structural geometry and connections should be simple to make construction easier and less expensive. Circular structures with fixed connections are preferred.
- Because of the structural load interaction, it is not possible to design a structural form exhibiting only compressive behavior. The upward force caused by the internal pressure on the roof will create in-plane tensile stresses and moments in the walls that should be controlled.
- Martian structures are first built and then pressurized. Initially, they should show appropriate behavior under dead load and then under internal pressure plus dead load, as the dominant Martian structural loading. In this study, the structures were initially analyzed under dead load and then under internal pressure plus dead load.
- The concentrated stress at the roof-wall and wall-surface connections should be controlled using practical methods.
- Relative optimization can be used to reduce the amount of material required for construction and improve the structural behavior of proposed models.

Abaqus which is a powerful finite element software for structural analysis was used for modeling and analysis^30^. The concrete damage plasticity model was used to simulate the behavior of concrete under applied loads and in accordance with the mechanical properties of simulated Martian sulfur concrete^30^. The model is a continuum, plasticity-based, damage model for concrete or brittle materials. The type of analysis was dynamic implicit analysis based on the unconditionally stable Hilber Hughes Taylor (HHT) time integration method using suitable fixed time increments^50,51^. A 10-node quadratic tetrahedron (C3D10) mesh element was used in accordance with the geometry of the structural models^30,52^. Internal pressure and dead load (as dominant Martian structural loads) were applied with proper order (“Extra notes and considerations” section) in the form of ramp pressure and acceleration magnitude, respectively. Additionally, it was assumed that structures were located on a rigid surface/rigid foundation (fixed supports/encastre boundary condition) (Supplementary Fig. 1).

## Extra notes and considerations

**Figure 17:**
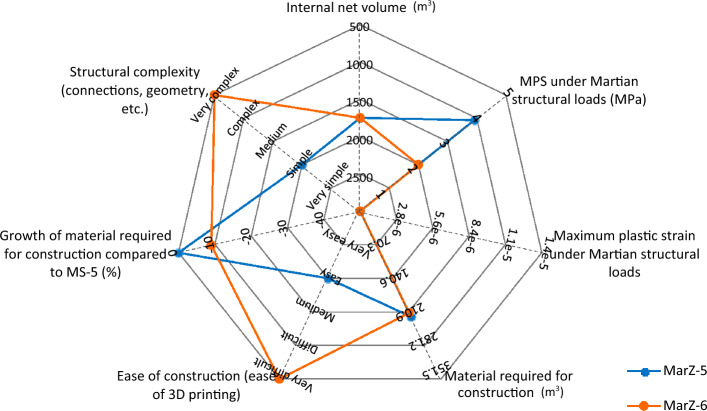
Multi-scale spider/radar chart for comparison of MarZ-5 and MarZ-6 in accordance with defined acceptance criteria and design limitations.

**Figure 18:**
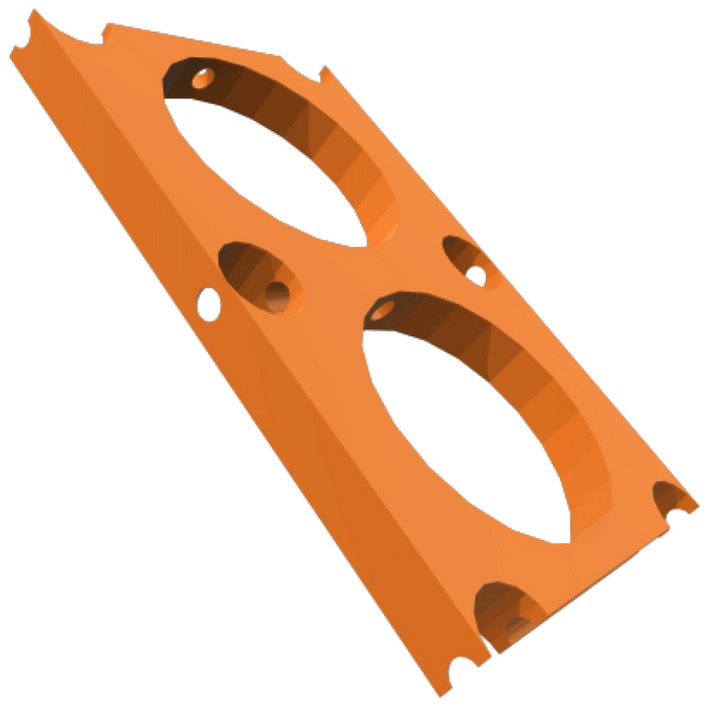
Interconnected cavities concept in the perforated layer (middle layer) of MarZ-6.

The calculations and analyses that were performed in this study indicate that there are additional considerations that should be taken into account. These are:

- The structure with middle perforated layer (MarZ-6; “Structure with middle perforated layer” section) showed the best structural behavior as well as the lowest amount of material required for construction compared to all other proposed Martian structures, including MarZ-1 to MarZ-5. This model included a wall with three layers, the middle layer of which was a perforated layer in a specific pattern. This model makes it a very complex model that is more difficult to construct compared to the other models. It would increase the probability of nozzle failure and the construction energy and cost should be considered at the time of construction. Because of these considerations, this study proposes model MarZ-5 as the best structural model. Figure 17 compares models MarZ-5 and MarZ-6.
- The settlement and colonization of Mars would be a long-term mission; thus, a human-friendly design will have an intensive effect on the mission’s success. Martian structures should approximate an environment similar to Earth; thus, GCRs and SEPs prohibit the use of glass (or similar material) for Martian structures. Also, glass would have to be imported from Earth at a high cost because the internal pressure of Martian structures would require thick and heavy panes. Considering the structural form of the proposed models, fiber optic cables could be used to provide light for residual and agricultural structures as a simple and low-cost solution^53^. The thin atmosphere of Mars assures good quality sunlight.
- Internal pressure is the most challenging force in Martian structural design. To preserve the health of the crew members and provide a human-friendly space in Martian structures, the internal pressure should be equal to the atmospheric pressure on Earth (101.325 kPa)^13^. Like on Earth, 21% of the air inside the structure should be oxygen. An alternative solution would be to decrease the internal pressure to 70 kPa and increase the oxygen to 26.5%^54^. This would make the design procedure easier. However, its feasibility, and effect on the health of the crew members and cost in the long term should be assessed under Martian conditions. It should be noted that the Martian structures proposed in this study were designed in accordance with 1 atm of internal pressure.
- A combination of Martian structural loads that produce worst-case scenarios during construction and operation should be considered for the design of the Martian structures^13^. This procedure was considered for the design of the proposed Martian structures. As stated (“Main concept, limitations and equation for design” section), the proposed structures should be stable under gravity loading during construction and gravity load (dead load) plus internal pressure (during operation).
- Soureshjani et al. proposed a 200 kg/m^2^ live load for the design of Martian residential structures^13^. The structures proposed in the current study are single-story structures; thus, a live load would be applied directly to the floor (surface) and can be neglected in the design process. The dominant Martian structural loads used were gravity (dead load) plus internal pressure.
- Considering the behavior of the proposed structures under Martian structural loads and the similarity of these loads with pressurized water tanks, the concept of the proposed structural form is also appropriate for such tanks on Mars. In the design of water storage tanks (non-pressurized), the proposed wall forms can be used.
- Diffusion phenomenon occurs when particles of one substance move from an area of high concentration (higher pressure in this case) to an area of lower concentration (lower pressure in this case)^55,56^. Because of the major difference in pressure between the internal (high concentration) and external environments (lower concentration) of the Martian structures (about 1 atm), the internal air will tend to move toward the outside of the structure (air leakage). It is possible to use the diffusion phenomenon to propose a low-cost solution for air leakage without importing materials from Earth by using the interconnected cavities in the perforated layer (middle layer) of MarZ-6 to pressurize this layer with 1 atm CO_2_ gas, which is the most abundant gas in the atmosphere of Mars. This would prevent the internal air of the structure from leaking into the atmosphere. As the bond length of CO_2_ is greater than that of O_2_ (134 to 121), it would be harder for CO_2_ to leak into the building. However, further calculations and experimental studies are required regarding this approach. Figure 18 shows the middle perforated layer of the MarZ-6 model. Additionally, the failure of the 3D printer and the extra energy required for the construction of this approach should be evaluated.
- As previously pointed out, Mars is an elemental-rich planet. Several kinds of concrete and cement can be crafted on Mars using in-place sources such as aluminate concrete, sulfur concrete (used in this study), Mg- and Si-based concrete, POP, OPC, and so on^16,57,58^. These concrete/cement show different properties, workability and curing behavior on Mars. But all of them show brittle behavior (chiefly in tension) and limited tensile strength which is the main idea of the current study (it should be noted that the construction cost and required energy using different types of concrete/cement should be considered). Additionally, there are some options such as natural fly ash, and additives (such as polymer fiber, steel fiber, nanoparticles) that can improve mechanical properties, preventing crack initiation, and crack propagation (“Defined structural acceptance criteria” section), decrease shrinkage and improve thermal behavior that is vital for structural design on Mars. However, the cost of production and importing from Earth should be calculated^58,59^. Other methods such as FRP bounding and confinement can also be considered^60,61^.
- As previously mentioned, the proposed structures are loaded in accordance with the Soureshjani et al. study^13^. In the current study, after the design of the structures under dominant loads (internal pressure + dead load), structures were analyzed under an appropriate temperature change to evaluate the effect of the thermal stress (${T}_{M}$). It is clear that because of the linear behavior, optimized form and the lower amount of this stress compared to other cases, the undesirable thermal stresses can be controlled by using a suitable factor of safety (FS) or scale factor (> 1). As a proposal, to decrease the undesirable effect of the extreme temperature changes on Mars and by considering the seasonal behavior of the red planet, structures can be built in cold seasons. In this case, the increase in temperature will cause compressive stress and the undesirable effect of the temperature changes will be minimized. However, this is a proposal and the effect of the extreme temperature changes on the connections, durability and permeability in long-temp should be tested in the future using a full-scale concrete-built structure.

### Supplementary Information

Supplementary Information.

## Data Availability

The datasets used and/or analyzed during the current study are available from the corresponding author on reasonable request.
