# Supplementary material for: Sustainable colonization of Mars using shape optimized structures and in situ concrete
Source: Sci Rep. 2023 Sep 21;13:15747. doi: 10.1038/s41598-023-42971-9 (PMC10514203; doi:10.1038/s41598-023-42971-9)
Supplement: Supplementary file 1 — Supplementary Information. [file 41598_2023_42971_MOESM1_ESM.docx]

**Supplementary Information**

**Sustainable colonization of Mars using shape optimized structures and in situ concrete**

Omid Karimzade Soureshjani ^1^, Ali Massumi ^1^* and Gholamreza Nouri ^1^

*^1^ Department of Civil Engineering, Faculty of Engineering, Kharazmi University, Tehran, Iran*

*Corresponding author, Professor of Structural Engineering, E-mail: massumi@khu.ac.ir

1. **Supplementary Information**
   1. Comparison of the behavior of MarZ-1 through MarZ-5

|   Martian Sulfur concrete tensile strength limit: 3.92 MPa | | |
| --- | --- | --- |
| (f) | | |
| MarZ-1 | MarZ-5 | MarZ-2 |
| MarZ-3 |  | MarZ-4 |
| **✝** According to the construction process for a Martian structure and sec. Main concept, limitations and equation for design, the gravity load (dead load) should be applied first and then the internal pressure (as the dominant structural loads of a Martian structure). The structure must be stable during this loading process. This is why the MPS curves did not start exactly at zero. | | |
| **Supplementary Fig. 1 \|** Maximum principal stress (MPS) curves of proposed Martian structural models under dominant Martian structural loads (dead load plus 1atm internal pressure) | | |

**Supplementary Table 1|** Structural behavior details of proposed models (MarZ-1 through MarZ-5)

| Martian structural model | MPS under gravity load (MPa) | MPS under Martian structural loads (gravity load + one-atmosphere internal pressure) (MPa) | Maximum Plastic strain under Martian structural loads | Internal net volume (m^3^) |
| --- | --- | --- | --- | --- |
| MarZ-1 | 0.10 | 3.92 | 0.00 | 1741.22 |
| MarZ-2 | 0.08 | 3.92 | 0.00 | 1732.22 |
| MarZ-3 | 0.13 | 4.01 | 1.41×10^-5■^ | 1700.11 |
| MarZ-4 | 0.06 | 3.92 | 0.00 | 1713.70 |
| MarZ-5 | 0.06 | 3.92 | 0.00 | 1698.08 |

■ Very little of plastic strain, so it was ignored.

|   -37.12%^▲^  -30.48%^▲^  -24.97%^▲^  -5.49%^▲^ |
| --- |
| ▲ increase in concrete required for construction of each structure compared to MarZ-1 |
| **Supplementary Fig. 2 \|** Comparison of Martian concrete required for construction |

| 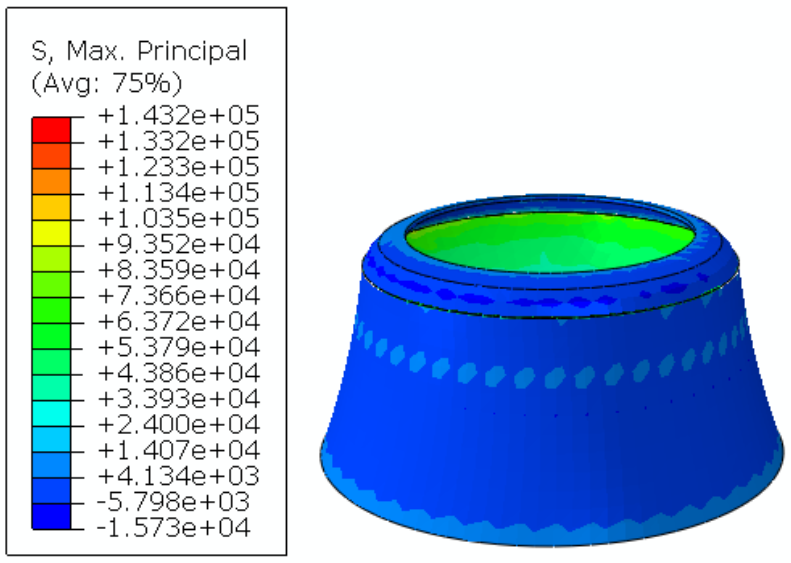 | 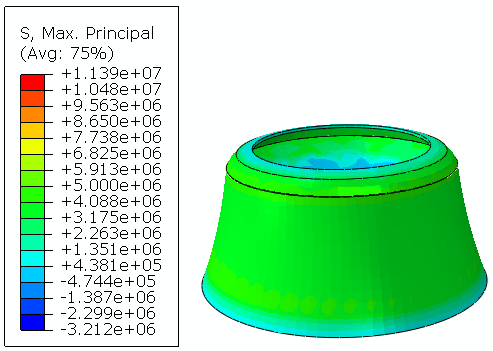 |
| --- | --- |
| **Supplementary Fig. 3 \|** behavior MarZ-5 (MPS contour) under dominant Martian structural loads (stability during construction and operation): **(a)** dead load; **(b)** dead load plus 1atm internal pressure | |

- 1. Structure with middle perforated layer

<https://drive.google.com/file/d/10isPzrYTOnUMRmUabTuqfhaU2xQ6JKyF/view?usp=sharing>

| 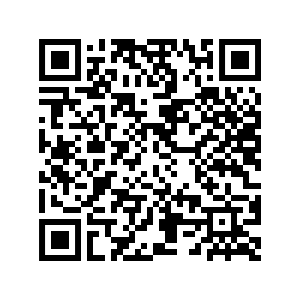 |
| --- |
| **Supplementary Video 1 \|** Middle layer of the perforated catenary model |

- 1. Design of a Martian structure using the shape optimization algorithm

|  |
| --- |
| **Supplementary Fig. 4 \|** Optimization process and convergence |

- 1. Conceptual reconstruct (3D printing)

<https://drive.google.com/file/d/1-l_6qqlzDV_oCrIG6TB4_9abbJrIRt43/view?usp=sharing>

| 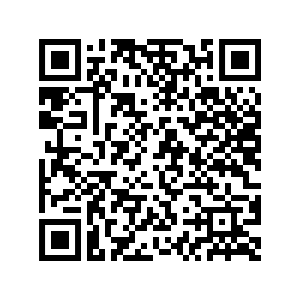 |
| --- |
| **Supplementary Video 2 \|** Printing process |
